# An intriguing case of lichen simplex chronicus in an elderly sub-Saharan African with longstanding scabies and sensory neuropathy

**DOI:** 10.11604/pamj.2019.34.124.19999

**Published:** 2019-11-01

**Authors:** Ekongefeyin Sintieh Nchinda Ngek, Ridley Mbwoge Nsioge, Michael Budzi Ngenge, Benjamin Momo Kadia

**Affiliations:** 1 Ekoumdoum Baptist Health Center, Yaounde, Cameroon; 2 Health and Human Development (2HD) Research Group, Douala, Cameroon; 3 Kumba District Hospital, Kumba, Cameroon; 4 Etougebe Baptist Hospital, Yaounde, Cameroon; 5 Foumbot District Hospital, Foumbot, Cameroon; 6 Grace Community Health and Development Association, Kumba, Cameroon

**Keywords:** Lichen simplex chronicus, scabies, neuropathy

## Abstract

Lichen Simplex Chronicus (LSC) is chronic dermatitis caused by repetitive scratching or rubbing of the skin. It presents as hyperpigmentation and thickening of the skin with variable scaling. Because LSC is a secondary lesion with a wide variety of causes, optimal management is contingent on identifying and managing its exact aetiology. We report an intriguing case of LSC in an elderly patient with longstanding scabies and sensory neuropathy.

## Introduction

Lichen Simplex Chronicus (LSC) is hyperpigmentation and thickening of the skin with variable scaling [1]. It is secondary to repetitive scratching or rubbing of the skin [2-5]. Chronic pruritus that leads to the development of LSC could be the consequence of diverse pathological processes such as inflammatory skin diseases, systemic diseases, neuropathic conditions, and even psychogenic disorders [3]. Thus, because LSC is a secondary skin lesion with a wide variety of causes, optimal management of this skin condition is contingent on the identification and treatment of its exact aetiology. This report describes an intriguing case of LSC in an elderly sub-Saharan African with longstanding scabies and sensory neuropathy.

## Patient and observation

**Figure 1:**
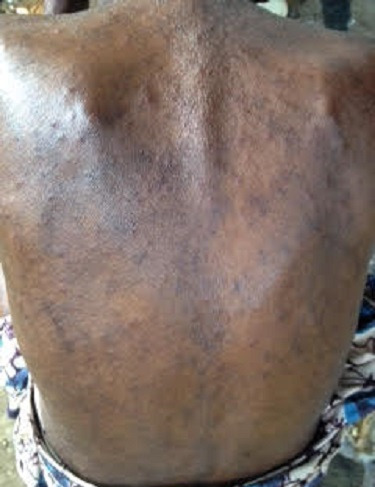
Skin lesions due to scabies on the patient´s back

**Figure 2:**
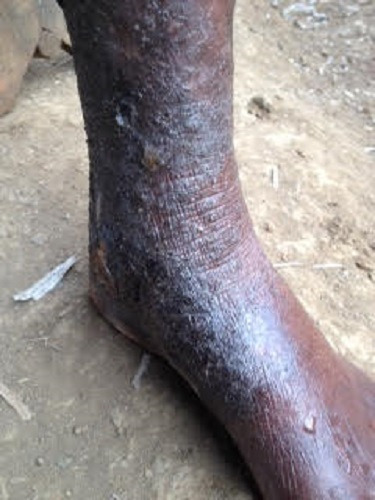
Lichen simplex chronicus on the left leg of the patient

In the course of a community outreach program in a remote village of Cameroon, we encountered a 70-year-old woman of black ethnicity who complained of intense pruritus, scarring and blackening of the distal left leg for 5 months. She also reported a progressive ulceration of the leg due to persistent scratching. Three months prior to the onset of the lesions on her leg, she had developed generalized pruritic vesiculopapular rashes which progressively became associated with excoriations. These rashes had started as erythematous papular lesions in her finger webs then spread to the arms, trunk, groins and lower extremities. The rashes were unsuccessfully treated as “filarial rash” in different health centers using suboptimal doses of ivermectin. It is worth noting that the patient had no rashes until 2 months prior to the onset of the generalized pruritic rashes when she began sharing her bed with her grandchildren who had similar rashes for which they had never received treatment. Her past history was negative for known allergies or known chronic illnesses like diabetes or chronic liver disease. She was a widow and lived alone in the village, depending on small scale subsistence farming. The review of systems was without particularity. On clinical examination, this elderly patient looked unhappy and her general hygiene was poor. She had obvious generalized vesicular rashes interspersed with crusted papular rashes and excoriations (Figure 1). Her vitals were normal. Her conjunctivae were pink and her sclera were anicteric. There was no lymphadenopathy. The anteromedial aspect of the distal third of her left leg had a thick black desquamating scaly papular lesion and a small healed ulcer (Figure 2). There were no palpable subcutaneous nodules. The distal half of her left leg had no sensation to pain, fine touch and crude touch. Neurological examination was otherwise normal. There was no pedal oedema and no varicosities. The peripheral pulses in her lower limbs were symmetrical and the ankle brachial pulse index was normal. The rest of the physical examination was normal as well. Our patient's historical and clinical data were suggestive of LSC of the left leg with probable aetiologies being long standing scabies and sensory neuropathy. We also considered emotional stress as a possible aetiological differential. Our differential diagnoses of LSC were lichen planus, prurigo nodularis, taeniasis and lipodermatosclerosis. However, lipodermatosclerosis was less likely in the absence of signs of venous insufficiency. Skin scrapings were taken from the borders of the crusted papular rashes and the lesion on the distal left leg.

These were successfully viewed directly under a light microscope coverslip with immersion oil and eggs of sarcoptes scabiei were observed; this confirmed the diagnosis of scabies. Ten percent (10%) potassium hydroxide was also applied on a sample of scrapings obtained from crusted papular rashes and viewed under the light microscope. This test was negative for fungal hyphae. Skin biopsy to rule out psoriasis, prurigo nodularis and lichen planus could not be done given the resource constraints. Also, nerve conduction studies to verify the type of the neuropathy our patient had could not be done. The patient's fasting blood sugar was measured serially to rule out diabetes and its possible complications which include sensory neuropathy. The fasting blood sugar values were normal. The following was our treatment plan: 1) Benzyl benzoate 25% cream: it was applied over the whole body, then reapplied without bathing on the following day and washed off 24 hours later. A third application was deemed necessary. 2) Oral ivermectin: 200 µg/kg/dose; 5 doses were given at days 1, 2, 8, 9 and 15. 3) Oral prednisolone: 2 mg/kg/day for 3 days, with the dosage gradually tapered over a period of 2 weeks to reach 0.1 mg/kg/day. 4) Oral chlorpheniramine: a total of 16 mg in four divided doses for 5 days. 5) Application of a vaseline-imbibed dressing on the distal left leg to keep the leg moist and occlude it from scratching. 6) Treatment of other infested family members. 7) Education of the family on hygiene and sanitation. The patient was adherent to the drug therapy and tolerated the drugs. After 8 weeks of follow-up and psychosocial support, there was significant regression of the skin lesions caused by LSC and scabies and the patient was referred for neurology consult for proper management of the sensory neuropathy.

## Discussion

LSC is a serious skin condition that considerably affects the patient's quality of life [1,2,6]. It begins as itchy skin. The itching leads to scratching and rubbing which causes the skin to become thickened and darkened. The thickened skin is itchier which causes more scratching and more skin thickening [1,7]. If not treated, the scratch-itch cycle continues and could cause significant physical injury such as ulceration whose onset and progression was possibly enhanced by sensory neuropathy in our patient. The historical and clinical data of the patient initially pointed to a diagnosis of LSC probably due to chronic pruritus induced by longstanding scabies. Scabies is a transmissible parasitic skin disorder caused by sarcoptes scabiei. The primary lesions of scabies include superficial linear burrows; inflammatory papules and nodules in the axilla and groin. The secondary lesions consist of small urticarial crusted papules, eczematous plaques and excoriations [8]. Advanced age and poor hygienic conditions as well as the contact history with scabies were risk factors for scabies in the case presented. It is worth noting that pathognomonic signs of scabies such as burrows may not be observed in a person with dark skin. More so, in an elderly patient, scabies may manifest as intense pruritus with only subtle skin findings [9]. These challenges made the clinical diagnosis of scabies equivocal in our case and therefore laboratory confirmation of scabies was imperative. There was some uncertainty in the aetiology of LSC in our patient because her clinical data was also suggestive of sensory neuropathy involving the leg where LSC had developed. There is evidence suggesting that neurological disorders such as neuropathy and radiculopathy which involve damage to the peripheral nervous system may be implicated in the aetiology of LSC. Some authors have even proposed that a diagnosis of LSC on a limb should warrant a search for an underlying neuropathy even if the patient has no symptoms of neuropathy [10]. These suggestions and the location of LSC on a neuropathic limb lend further credence to sensory neuropathy as the cause of LSC in our patient with the possibility of longstanding scabies being a red herring.

It is worth mentioning that although sensory neuropathy and longstanding scabies appeared to be the most obvious aetiological differentials of LSC, the possibility of chronic pruritus and LSC being associated with emotional stress due to the patient's family and socioeconomic condition was also explored. This is justified by recent evidence suggesting that psychological stress is associated with the development and exacerbation of LSC [2,5]. Although LSC can conveniently be diagnosed clinically (a fully developed plaque has an outer zone of discrete, brownish papules and a central zone of scaly confluent papules), the inability to perform skin biopsy and nerve conduction studies prevented us from ruling out skin disorders similar to LSC and to verify the aetiology of the sensory neuropathy respectively. In spite of the gradual regression of the skin lesions following our management plan, the cause of the neuropathy was still not elucidated. In all, the exact aetiology of LSC in our patient remained unclear and if neuropathy or a psychosocial problem was at the origin of LSC, then our patient would be at risk of resurgence of pruritus and LSC. Nonetheless, we emphasized on psychosocial support and the need for neurology consult in our management. The mainstay of treatment of LSC is patient education about the effects of scratching. Secondary treatment is with topical corticosteroids and in some cases intralesional injections of long-acting corticosteroid. Alternatively, a tape impregnated with flurandrenolide may be preferred because occlusion prevents scratching [9]. A particularity in the case presented is that given the resource constraints, we relied on the use of a less sophisticated treatment plan and we recorded satisfactory results. Apart from the problems of poor cosmesis and sleep disturbances with possible depression, it is worth noting that the plaques of LSC are characterized by squamous cell hyperplasia and dermal scarring with a small risk of progression to squamous cell carcinoma [11]. This further indicates the need for prompt and optimal management of LSC.

## Conclusion

LSC is a serious and debilitating skin disorder resulting from chronic pruritus due to an underlying disease process. Albeit an unusual and perplexing phenomenon, a patient could have multiple plausible aetiologies for LSC and this should be verified even amid resource constraints in order to achieve optimal management and limit the diverse complications linked with LSC.

## Competing interests

The authors declare no competing interests.
